# Coffee Bean and Its Chemical Constituent Caffeine and Chlorogenic Acid as Promising Chemoprevention Agents: Updated Biological Studies against Cancer Cells

**DOI:** 10.3390/molecules29143302

**Published:** 2024-07-12

**Authors:** Mohamed Aborziza, Riezki Amalia, Ade Zuhrotun, Nur Kusaira Khairul Ikram, Dhania Novitasari, Muchtaridi Muchtaridi

**Affiliations:** 1 Department of Pharmaceutical Analysis and Medicinal Chemistry, Faculty of Pharmacy, Universitas Padjadjaran, Sumedang 45363, Indonesia; mohamed22002@mail.unpad.ac.id (M.A.); dhania@unpad.ac.id (D.N.); 2 Department of Pharmacology and Clinical Pharmacy, Faculty of Pharmacy, Universitas Padjadjaran, Sumedang 45363, Indonesia; riezki.amalia@unpad.ac.id; 3 Department of Biology Pharmacy, Faculty of Pharmacy, Universitas Padjadjaran, Sumedang 45363, Indonesia; ade.zuhrotun@unpad.ac.id; 4 Institute of Biological Sciences, Faculty of Science, Universiti Malaya, Kuala Lumpur 50603, Malaysia; nkusaira@um.edu.my; 5 Research Collaboration Centre for Radiopharmaceuticals Theranostic, National Research and Innovation Agency (BRIN), Jln. Raya Bandung Sumedang Km. 21, Sumedang 45363, Indonesia

**Keywords:** coffee, caffeine, chlorogenic acid, chemopreventive, autophagy, tumor cells

## Abstract

Cancer is a complicated and ever-evolving disease that remains a significant global cause of disease and mortality. Its complexity, which is evident at the genetic and phenotypic levels, contributes to its diversity and resistance to treatment. Numerous scientific investigations on human and animal models demonstrate the potential of phytochemicals in cancer prevention. Coffee has been shown to possess potent anti-carcinogenic properties, and studies have documented the consumption of coffee as a beverage reduces the risk of cancer occurrence. The major secondary metabolites of coffee, named caffeine and chlorogenic acid, have been linked to anti-inflammatory and antineoplastic effects through various signaling. In light of this, this review article provides a comprehensive analysis based on studies in anticancer effects of coffee, chlorogenic acid, and caffeine published between 2010 and 2023, sourced from Scopus, Pubmed, and Google Scholar databases. We summarize recent advances and scientific evidence on the association of phytochemicals found in coffee with a special emphasis on their biological activities against cancer and their molecular mechanism deemed potential to be used as a novel therapeutic target for cancer prevention and therapy.

## 1. Introduction

The development of novel and effective cancer medicines is critical, given the global expanding incidence of malignant diseases. The multifaceted nature of cancer, as evidenced at both the molecular and clinical levels, highlights its diversity and treatment resistance [1]. Despite the obstacle in developing cancer chemopreventive agents based on natural sources, there are still several promising pieces of evidence that support the evaluation of potential active natural products with regards to reducing or reversing the premalignant tissues [2]. Furthermore, the notion of cancer chemoprevention surfaced from anecdotal experience with nutritious meals, as well as epidemiological studies, with most of that focused on cancer treatment. For instance, people who consume plant-based foods are thought to have a lesser risk of cancer, showing increased interest in dietary phytochemical studies [3].

Coffee, being one of the most widely consumed beverages worldwide [4], has been shown to contain potent natural chemopreventive and antineoplastic agents [5,6]. Coffee is derived from the berries of the *Coffea* sp., but only two species were considered for production, including *Coffea arabica* (Arabica) and *Coffea canephora* (Robusta). Apart from its renowned effect as a stimulant due to the high amount of caffeine, the bioactive compounds in coffee have been increasingly explored for other biological activities, from antioxidant to associated activities, including antiinflammation and anticancer [7]. These occurrences have caught the attention of health experts, given that coffee consumption has rapidly expanded over the last few decades due to greater prosperity and economic interest [8,9].

The chemical constituents of coffee beverages are mostly determined by the processing procedures (pre-roasting and roasting) used to prepare green coffee beans. Furthermore, harvesting methods and industrial processes for green coffee, as well as consumer ways for preparing coffee beverages, all contribute to variations in the concentration of particular substances in the final product [10]. Various factors such as coffee species, growing circumstances, harvesting methods, and processing procedures (such as high-temperature roasting) affect the amount of bioactive chemicals in coffee, such as antioxidants and biogenic amines [11,12]. Coffee beans comprise an abundance of xanthine-based caffeine, polyphenol chlorogenic acids, and tannins [10], followed by other polyphenols and flavonoids which possess the antioxidant properties [13]. Numerous epidemiological studies have demonstrated that coffee consumption has been associated with potential health advantages due to its anti-inflammatory and chemopreventive activities. It has been proposed that the antioxidative properties of several coffee ingredients are responsible for the decrease in inflammation when coffee is administered [14]. We therefore sought to review the recent advances and knowledge in the association of major phytochemicals present in coffee (caffeine and chlorogenic acid), with their preventive or therapeutic effects targeted at the cellular and molecular mechanisms that lead to cancer progression.

## 2. Biochemistry and Metabolism of Caffeine from Coffee Beans

**Figure 1:**
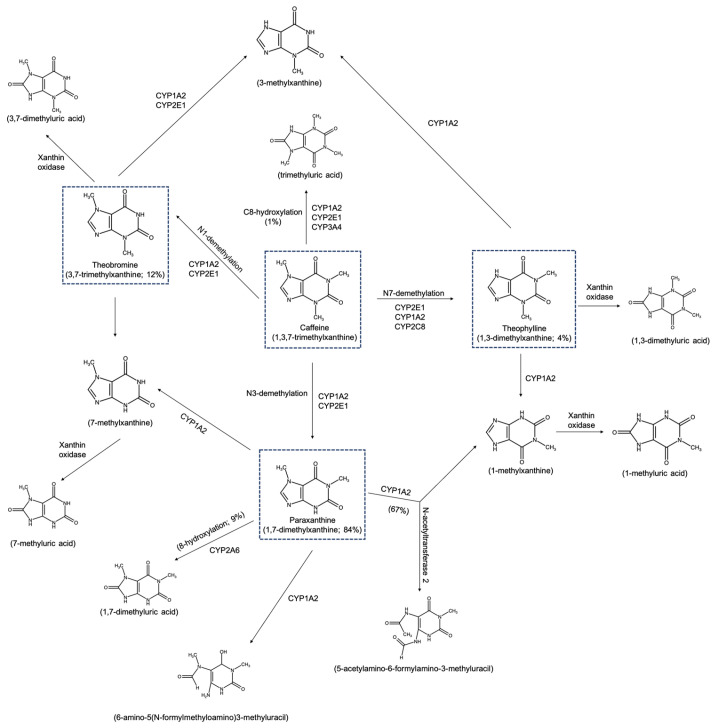
The major metabolism of caffeine in humans, adopted from [31].

Around 1.67% of dried green coffee contains caffeine (1,3,7-trimethylxanthine) regardless of the different geographical origins that would affect the amount of caffeine [15]. Concerning oral consumption of caffeine in beverages, the caffeine is mostly absorbed in the gastrointestinal tract and small intestine, with unnoticeable significant first pass effect. Following absorption, caffeine spreads swiftly throughout plasma-binding. It has been found to occur in bile, saliva, semen, breast milk, and umbilical cord blood. The caffeine-plasma concentration peaks between 15 and 120 min after oral consumption. Notably, after ingestion, caffeine may rapidly pass through cell membranes with detectable levels in the brain as early as 5 min [16]. The study by Lin et al. [17] showed that daily caffeine intake affected higher concentrations of caffeine in gray matter and cerebral blood flow, indicating the accumulation of caffeine residual in the brain. The primary metabolism of caffeine occurs in the liver through phase-I oxidation by cytochrome P450 1A2 resulting in active paraxanthine as a major metabolite, followed by theobromine and theophylline [18,19,20,21,22] (Figure 1). Prior reports have already discovered the connection between daily coffee consumption and caffeine metabolism through the polymorphism of CYP1A2 and CYP2A6 [23,24]. The second phase conjugated-metabolism produces a mixture of di- and tri-methylated xanthine, uric acid, and acetylated uracil derivatives, all being excreted through urine [25]. Previous studies have established that the biological effects of caffeine are tightly associated at three primary modulatory points: an antagonistic action on adenosine receptors, calcium mobilization, and phosphodiesterases inhibition [26,27].

The capacity of caffeine (and metabolite paraxanthine) to inhibit adenosine receptors due to their similar purine structure, shows its significant effect regarding cellular energy and inflammatory response [28,29]. Furthermore, caffeine induces intracellular activity on calcium and the cyclic adenosine monophosphate phosphodiesterase (cAMP) pathway by inhibiting phosphodiesterase in adipose tissue and skeletal muscle [30], resulting in cardiostimulatory and antiasthmatic actions [31]. Adenosine receptor stimulation leads to an increase in cAMP production, which may reduce the inflammatory response in a variety of pathophysiological circumstances. Despite caffeine not being a selective adenosine receptor antagonist, its modulatory effects on adenosine receptors may worsen the acute inflammatory response which depends on its concentration [32,33]. Additionally, caffeine stimulates calcium release by activating ryanodine receptors in skeletal muscles, raising intracellular calcium and speeding up the excitation–contraction coupling process, thus playing a crucial role in the neurotransmitters released by neurons [34,35]. Recent studies of caffeine also documented several mechanisms that involve systemic metabolism and oxidative-inflammatory signaling, indicating that caffeine also affects peripheral signaling and may have beneficial effects on the human body regarding the aging process [16].

## 3. Biochemistry and Metabolism of Chlorogenic Acid from Coffee Beans

**Figure 2:**
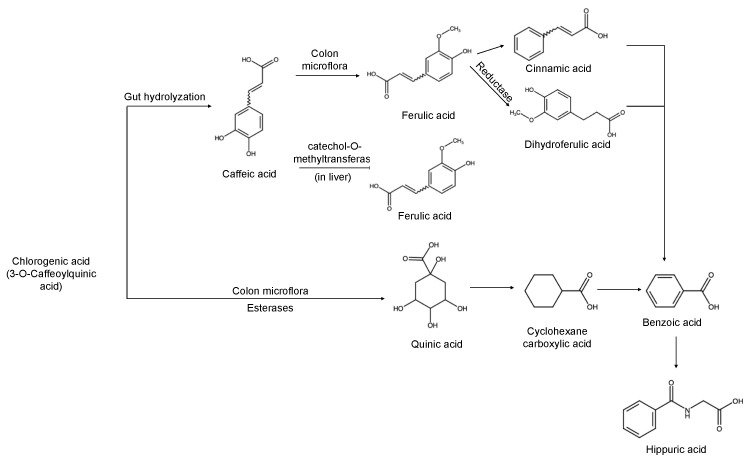
The major metabolism of chlorogenic acid in humans, adopted from [37,40].

There is a greater amount of chlorogenic acid (CGA) in green coffee bean than caffeine (5.43%), and this would be lost during roasting [15,36]. Most of the biotransformation of chlorogenic acids in humans occurs in the colon, followed by the liver [37]. Dietary chlorogenic acids are absorbed in the small intestine, next they are hydrolyzed with esterases from gut mucosa into quinic acid and caffeic acid (Figure 2), and then they pass into the bloodstream. A substantial amount of the unaltered chlorogenic acid enters the colon, where it is metabolized by esterases produced by colon microflora. The colon plays a crucial role in transforming both caffeic and ferulic acid into dihydroferulic acid and facilitating their absorption through the intestine. Caffeic acid, e.g., 3, 4-dihydroxycinnamic acid, is converted by the enzyme catechol-O-methyltransferase into another phenolic acid, ferulic acid [38]. Both compounds have the ability to form an ester bond with quinic acid, resulting in the formation of various isomers within the chlorogenic acid family. Most of the metabolized products from chlorogenic acid result from reaction with transferase, and are excreted as another form of benzoic acid called hippuric acid [39,40].

## 4. The Role of Coffee in Chemoprevention Activities on Carcinogenesis

The targeted molecular pathways for developing and accessing future cancer-management techniques are carcinogenesis and chemoprevention. Chemoprevention refers to the use of pharmaceutical methods to stop or reverse the development of cancer before invasion and/or metastasis take place. According to epidemiological research, coffee consumption may be associated with a lower risk of cancer. The potential role of coffee in cancer chemoprevention has been supported by a number of experimental models, including human [41,42]. The scientific literature has hypothesized a variety of coffee-dependent mechanisms, including the suppression of oxidative stress and damage, the activation of metabolizing liver enzymes involved in the detoxification processes of carcinogens, and modulation of the inflammatory response. Furthermore, specific coffee ingredients have been shown to affect tumor cell apoptosis, proliferation, and metastasis, and to exhibit anti-angiogenic properties [43,44]. Interestingly, a higher intake of decaffeinated coffee significantly reduced the risk of colorectal cancer, but this effect was not observed with caffeinated coffee. However, it is known that caffeinated coffee can lower the risk of rectal tumor [45]. In another cohort study, both caffeinated and decaffeinated coffee consumption improved overall survival (OS) and progression free-survival (PFS) in patients with metastatic colorectal cancer [46]. Furthermore, frequent consumption of all coffee types lowered the chance of liver disease and carcinoma [47,48], while daily coffee intake reduced tumor size in invasive breast tumor with positive estrogen receptor (ER) more effectively than in triple-negative tumor [49]. These findings suggest that while drinking coffee with or without caffeine provides equivalent health benefits, caffeine may still play a role in some coffee-induced effects which also likely depend on the subsite of the tumor [50].

In addition to the chemopreventive activities demonstrated by caffeine and chlorogenic acid, studies have indicated that coffee extracts and kahweol also possess anti-carcinogenesis properties in a number of cancer cell lines. Kahweol inhibits cancer growth in macrophage cells of mice via activating the NF-κB pathway [44,51,52]. Moreover, co-treatment with kahweol and cafestol has demonstrated anti-carcinogenic effects in male F344 rats [53]. Additionally, kahweol and cafestol together have been observed to provide chemoprevention against malignancies caused by heterocyclic amines. Given that coffee constituents have the potential to exhibit antioxidant, cytotoxic, anti-mutagenic, and carcinogenic properties, they are therefore being studied for the treatment of different types of cancer, with particular attention to cafestol and kahweol [54,55], as these compounds may serve as valuable supplements to cancer prevention or therapy.

## 5. The Antitumor Activities of Coffee and Its Chemical Constituent

A substansial report related to coffee extract and its metabolite constituents in cancer cells is summarized in Table 1. Caffeine directly inhibits the cyclin D/CDK 4/6 complex which causes G1 arrest independently of p53 [49], and several reports also revealed that caffeine overrides the G2 phase arrest caused by DNA-damaging chemicals, propelling the cells into a deadly mitosis. Caffeine’s capacity to restart Cdc25C and Cdc2 activity contributes to averting G2 arrest [56,57]. Interestingly, due to its planar xanthine structure, caffeine is hypothetically a formed π-π complex with nucleobases in DNA [58], which is similar to conventional anticancer drugs [59]. In addition to triggering DNA intercalation, a report by Moura et al. [58] found that caffeine had two possible roles: to protect DNA against DNA-damaging agents and to modulate intercalating drugs used in chemotherapy treatments.

Previous studies have documented that in melanoma cells, caffeine has a modulatory effect on the signaling cascades of AMP-activated protein kinase (AMPK), PI3K/Akt, and the mammalian target of rapamycin (mTOR) [60]. Moreover, caffeine downregulates the expression of several proteins, including retinoblastoma protein (Rb), extracellular signal-regulated kinases (ERK) 1/2, GSK3β, pyruvate dehydrogenase kinase 1 (PDK1), cyclin D1, cyclin E, c-Myc, Akt, and mTOR in various cancer cell lines [61,62,63]. In another study, caffeine upregulates p300 expression in glioma cells [64]. Caffeine has been observed to reduce the phosphorylation of ERK induced by NF-κB in osteoclasts. A similar phenomenon also occurred in macrophage RAW 264.7 to suppress pro-inflammatory genes following lipopolysaccharide (LPS)-induced inflammation [62,65]. In addition, coffee also demonstrated antitumor activity in vivo [66,67], and several studies have been conducted in humans to assess the correlation of coffee consumption and the risk of cancer [68].

Chlorogenic acid in coffee has demonstrated antitumor action against cancer cell lines by reducing cell survival and suppressing reactive oxygen species (ROS) [69]. Additionally, it has been observed to suppress the production of cell adhesion molecules in human endothelial cells that are triggered by TNF-α16 [70]. Cafestol possesses anti-angiogenesis action in human umbilical vein endothelial cells, as it inhibits the proliferation, migration, and tube-formation ability of the cells [71,72]. Ferulic acid also inhibits angiogenesis via targeting FGFR1 and activating the PI3K/Akt signaling pathways, limiting cell proliferation via cell cycle arrest and death in addition to reducing invasion, migration, and colony formation [73,74]. Kahweol in green coffee bean has been shown to have an anti-angiogenic impact in zebrafish and chicken chorioallantoic membranes, in addition to exhibiting other significant activities including cell cycle arrest, anti-angiogenesis/proliferative, and associated phenomena [75].

Currently, numerous cytotoxic medicines are utilized clinically for the treatment of various cancer types, despite their substantial side effects, low rate of cure, and development of resistance. Coffee, owing to its widespread availability, low cost, and racial compatibility, may hold promise as a significant anti-cancer treatment option [50]. In addition, combining coffee constituents (caffeine or chlorogenic acid) with existing chemotherapeutic drugs in cancer therapy has been evaluated. The combination of caffeine with doxorubicin prevented the efflux effect of doxorubicin from cancer cells and enhanced the cytotoxic activity [76]. A similar result was demonstrated in the synergistic effect of caffeine in cisplatin-treated sarcoma tumors [77]. Other antitumor drugs have also been summarized in a review by Ialongo et al. [78]. Clinical trials for caffeine have been reported in several publications, mainly combined with DNA-intercalating agents including cisplatin, doxorubicin, and the tyrosine kinase inhibitor dovitinib [79,80,81].

**Table 1:** The cytotoxic activities of chemical constituents of coffee bean against cancer cells.

Compound	Concentration	In Vitro Model	Mechanism of Action	Reference
Chlorogenic acid	10 µM	Human umbilical vein endothelial cells	Reduction in wound cell migration, cell invasion, hypoxia-induced tube formation	[82]
	25 and 50 µM	Glioma, lung cancer, colon cancer and solid tumor cell lines from hepatoma	Induction of cell differentiation, inhibition of cell proliferation, decreased expression of genes associated with poor differentiation, increased expression of key genes associated with differentiation	[83]
	5 mM	Leukemia (K562 cells)	Induction of apoptotic topoisomerase−DNA complexes and generation of hydrogen peroxide	[84]
	1–1000 µM	Liver cancer (HepG2 cells)	Inhibition of invasion and migration, inhibition of cell proliferation and colony formation, induction of cell death, decreased MMP2/TIMP-2, DNA methyltransferase1, ERK1/2 phosphorylation and MMP-9 expression, increased p53 and p21	[85,86]
	1–5000 µM	Lung cancer (A549 cells)	Inhibition of phorbol-12-myristate-13-acetate Stimulated invasion of A549 cells, induction of apoptosis, inhibition of cell proliferation, decreased stem cell marker related genes (CD44, NANOG, POU5F1, and SOX2), MAPK and PI3K/Akt signaling, inactivation of NF-κB, activator protein 1 and STAT3, hypoxia-induced HIF-1α protein level, transcriptional activity of HIF-1α, vascular endothelial growth factor and Bcl-2, increased Bax, Bax/Bcl-2, p38, JUN, and caspase 3	[69,75,82,84]
	20–200 µM	Irradiated plasmids	Decreased DNA single-strand breaks	[87]
Caffeine	0.5, 1, 2 mM	Prostate cancer (PC-3 and DU145 cells)	Inhibition of cell adhesion and motility and decreased cell proliferation	[88]
	0.1–5 mM	Breast cancer (MDA-MB-231, Tam-R, MCF-7 cells)	MDA-MB-231 cells: inhibition of cell proliferation by 40% MCF-7 cells: inhibition of cell proliferation by 80%, induction of cell death, decreased estrogen receptor, poly (ADP-ribose) polymerase cleavage, decreased cyclin D1, Akt and Bcl-xL, increased caspase 7, Tam-R cells: inhibition of cell proliferation	[49]
	50–400 µM	Irradiated plasmids	Decreased DNA single-strand breaks	[87]
	10–1000 mM	Liver inflammation (human hepatic stellate cells)	Decreased procollagen type Ic, alpha-smooth muscle actin expression and progression of intrahepatic induction of apoptosis, increased F-actin and cyclic adenosine monophosphate, fibrosis	[89]
	0.1–4 mM	Leukemia (NB4 cells)	Bax, increase p21 and caspase 3, induction of apoptosis, inhibition of cell proliferation	[90]
	2 mM	Lung cancer (HTB182 and CRL5985 cells)	Increase PUMA (CRL5985), inhibition of cell proliferation	[91]
Cafestol	1–40 µM	Renal cancer (Caki cells)	Induction of apoptosis, inhibition of proliferation, increased Bim, Bax and FADD-like IL-1β-converting enzyme)-inhibitory protein, increased caspases 2 and 3, cleavage of poly (ADP-ribose) polymerase, decreased Akt phosphorylation, Mcl-1, Bcl-xL, release of Cytochrome c and Bcl-2	[92]
	40, 80, 150 µM	Leukemia (HL-60 and KG1 cells)	Decreased ROS generation and clonogenic potential, increased caspase 3, CD11b and CD15 differentiation markers, induction of apoptosis	[43]
Kahweol	1–25 µM	Human umbilical vein endothelial cells	Decreased MMP-2 expression, urokinase, cyclooxygenase-2 and monocyte chemoattractant protein-1, inhibition of tubule formation, inhibition of cell proliferation, inhibition of migration, inhibition of invasion	[93]
	40 µM	Liver inflammation (primary Kupffer cells and primary hepatocytes)	Decreased lipopolysaccharide-stimulated phospho-nuclear factor kappa B and signal transducer and activator of transcription 3 expression and lipopolysaccharide-induced production of interleukin 1 alpha, interleukin 1 beta, interleukin 6, and tumor necrosis factor alpha	[94]
	0.1–10 µM	Leukemia (U937 cells)	Decreased Bcl-2, Bcl-XL, Mcl-1, XIAP and Akt phosphorylation, increased JNK pathway, JNK, ROS generation and caspases 2, 3, 8, and 9, cytochrome c release, inhibition of cell proliferation, induction of apoptosis	[95]
	1–200 µM	Colorectal cancer (HCT116, SW480, LoVo, HT-29 cells)	Decreased heat shock protein 70, Bcl2 and phosphorylated Akt, increased ATF3 transcription and caspase 3, poly (ADP-ribose) polymerase cleavage, induction of apoptosis	[51,96]
	10–90 µM	Lung cancer (NCI-H358, NCI-H1299 cells)	Inhibition of cell proliferation, induction of apoptosis, increased p21 and Bax, decreased cyclin D1, basic transcription factor 3, ERK signaling pathway and Bcl-2, Bcl-xL	[97]

## 6. The Role of Coffee in Inducing Apoptosis toward Cancer Cells

**Figure 3:**
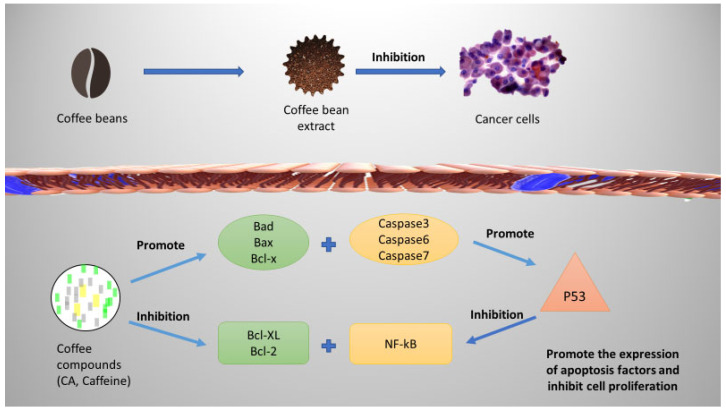
The hypothesized mechanism of coffee and its chemical compounds caffeine and chlorogenic acid of mediating apoptosis in cancer cells.

Coffee induces apoptosis by altering a number of the apoptotic response’s constituent parts (Figure 3). Different coffee compounds may target different apoptotic signaling mechanisms, such as increased cleavage of poly ADP ribose polymerase, downregulation of the signal transducer and activator of transcription 3 (STAT3) signaling pathway, and upregulation of the cyclic AMP-dependent transcription factor ATF3 [96,98]. Caffeic acid has been shown to produce apoptotic cell death and dramatically decrease Akt signaling in PC-3 human prostate cancer cells, TW2.6, and HCT 15 colon cancer cell lines. Additionally, it has been proposed to decrease congenic survival and apoptotic cell death in SCC25, CAL27, and FaDu cell lines [99]. Numerous studies suggest that chemical constituents in coffee may possess apoptotic potential. The antioxidant function of these substances is also influenced by their environment. Some mechanisms of action include the inhibition of ROS generation and pro-survival gene expression, conformational changes in pro-apoptotic proteins, loss of the mitochondrial membrane that activates caspases, and transcription factor Sp1 [100].

Despite its activity with respect to triggering apoptosis, it was later found that caffeine intake should be avoided in colorectal tumors treated with cell cycle modifying agents such as paclitaxel [101]. This was confirmed by Xu et al. who described that caffeine interferes with the anticancer effect of the antimitotic drug paclitaxel by preventing ⍺-tubulin acetylation, which could enhance the progression of lung and cervical tumors [102]. It is important to note that the effect of caffeine in preventing the cytotoxicity of chemotherapy can be associated with cancer type, as caffeine enhanced the apoptosis in paclitaxel-induced breast cancer cells [103]. Nevertheless, these reports suggest that patients receiving antimitotic drugs as part of their cancer therapy regimen should avoid consuming foods or beverages containing caffeine.

## 7. The Role of Coffee in Autophagy Process in Cancer Cells

A double-membrane autophagosome is formed as part of the intracellular breakdown process known as autophagy. This mechanism facilitates the removal of inclusion bodies and misfolded cytotoxic proteins more effectively than apoptosis [104,105]. Apart from programmed cell death, autophagy-induced cancer may also involve phosphatidylinositol 3-kinase (PI3K) pathways and the endoplasmic reticulum (ER) stress response. The dysregulation in this pathway has been linked to the development of cancer and resistance to cancer treatment, and it may have an impact on the level of autophagy in tumor cells [106,107]. In the same way, mTOR has also been identified as an autophagy mediator that contributes to cell growth, survival, and proliferation [108,109]. Furthermore, the abnormal relationship between autophagy, inflammation, and oxidative stress may aid in the development of innovative pharmacotherapeutic approaches for the management and treatment of cancer. Recent developments have proposed that induced autophagy is a novel target for cancer treatment [110,111].

It has been acknowledged that caffeine is able to suppress mTORC1 in both mice and in vitro models, to promote autophagosome generation in HepG2 cells, leading to the reduction of intracellular fats, to enhance β-oxidation, and to control hepatosteatosis [112,113]. It is noteworthy that caffeine in coffee has also exhibited cytoprotective effects in transformed skin cells, preventing cellular senescence and suppressing ROS generation by inducing SIRT3/AMPK-mediated autophagy [114]. Despite its initial approach in normal tissues, many studies have proven that inducing autophagy in cancer cells can be beneficial for chemotherapy agents with respect to eliminating cancer cells. A study by Erzurumlu et al. [115] showed that the addition of caffeine in docetaxel-treated breast cancer cells activated the unfolded protein response (UPR)-associated pathway and accelerated autophagy signaling due to increased Beclin-1 protein; this led to apoptosis in cancer cells as detected by the cleaved effector caspase-3. Also, methylxanthines derivatives (theophylline and caffeine) activated autophagy signaling through PTEN activation, followed by mTOR suppression in gastric tumor cells [116]. These findings open up the new challenge in caffeine development of inducing autophagy to initiate apoptosis in tumor cells, necessitating further experimental and clinical studies [117].

## 8. Conclusions and Recommendations

Coffee has been shown to exhibit anticancer activities through several mechanisms and shows its potential to prevent carcinogenesis. Among these mechanisms, coffee and its major content caffeine have promising activities regarding autophagy, which serves as a potential therapeutic target due to its strong association with other mechanisms such as cellular senescence and ROS production. Further investigations are warranted to answer several interesting questions regarding the specific mechanisms by which chemical compounds in coffee induce autophagy. Additionally, the consumption of coffee as a daily beverage also shows reduced risk of cancer occurrence, thus supporting further exploration of the supplementation of coffee as a chemopreventive agent for cancer. One thing to note is that the physiological characteristics of whole coffee will probably vary since coffee is a complex, non-standardized beverage, despite its remarkable results from in vitro and in vivo investigations utilizing certain coffee secondary metabolite constituents, which have shown a variety of biological activities. As a result, the bioactivity of coffee in mixtures can be possibly affected by matrix, synergistic, and/or antagonist effects in preventing tumor progression. Also, only a small proportion of the substances consumed can pass through the circulatory system and enter the tissues, and very little of the absorbed content may retain the original structure of the phytochemical from coffee. For these reasons, it is noteworthy that the prevention of many diseases prompted by coffee use is usually the result of the combined action of numerous components, and in some cases, the synergistic effect of multiple types of compounds is substantially superior to the activity of single compounds.

## Data Availability

There are no data outside that reported in this article.
